# Supplementary material for: The APPL1-Rab5 axis restricts NLRP3 inflammasome activation through early endosomal-dependent mitophagy in macrophages
Source: Nat Commun. 2021 Nov 17;12:6637. doi: 10.1038/s41467-021-26987-1 (PMC8599493; doi:10.1038/s41467-021-26987-1)
Supplement: Supplementary file 1 — Supplementary Information [file 41467_2021_26987_MOESM1_ESM.pdf]

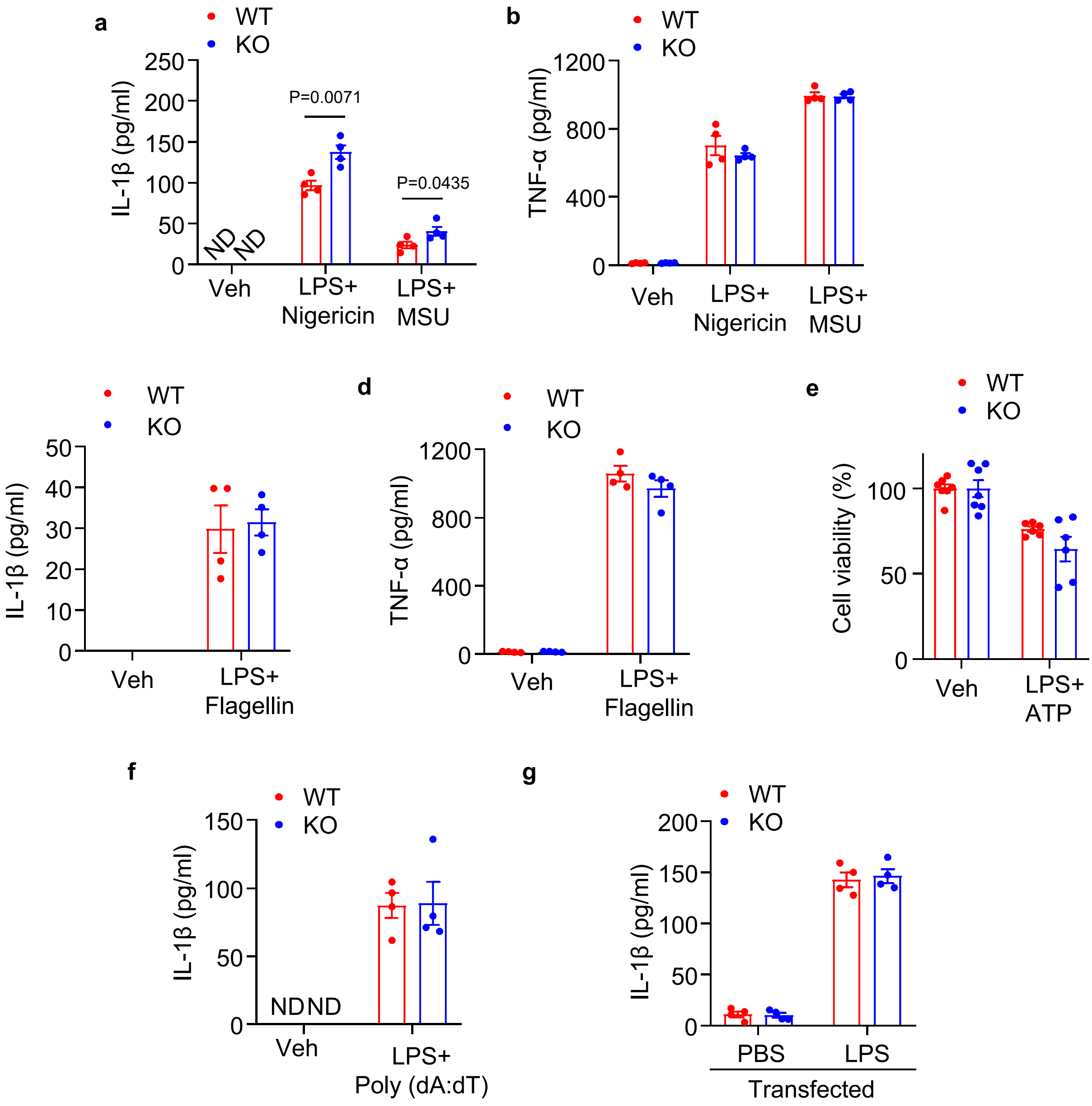

Supplementary figure 1. Effect of APPL1 deficiency on IL-1β production induced by diverse inflammasome activators. BMDM from APPL1 KO mice and their WT controls were primed with LPS (100 ng/ml) for 20 hours, followed by treatment with various inflammasome inducers including nigericin, monosodium urate crystals (MSU) or flagellin for 4 hours, 24 hours or 8 hours, respectively. (a and c) IL-1β and (b and d) TNF-α in cell culture supernatant were measured by immunoassays. n=4 biologically independent samples. (e) Lactate dehydrogenase levels released by the BMDM were measured and expressed as % of cell viability. WT-Veh and KO-Veh: n=7; WT-LPS+ATP & KO-LPS+ATP: n=6 biologically independent samples. (f) LPS-primed BMDM were transfected with poly (dA:dT) at 1 µg/ml using lipofectamine 3000 and incubated for 6 hours. n=4 biologically independent samples. (g) 2 µg/ml ultrapure LPS was delivered to BMDM using lipofectamine 3000 for 6 hours. (f-g). IL-1β level in the cell culture medium. n=4 biologically independent samples. Data are displayed as mean ± SEM. Statistical significance was tested using two-tailed student's *t*-test (a-g).

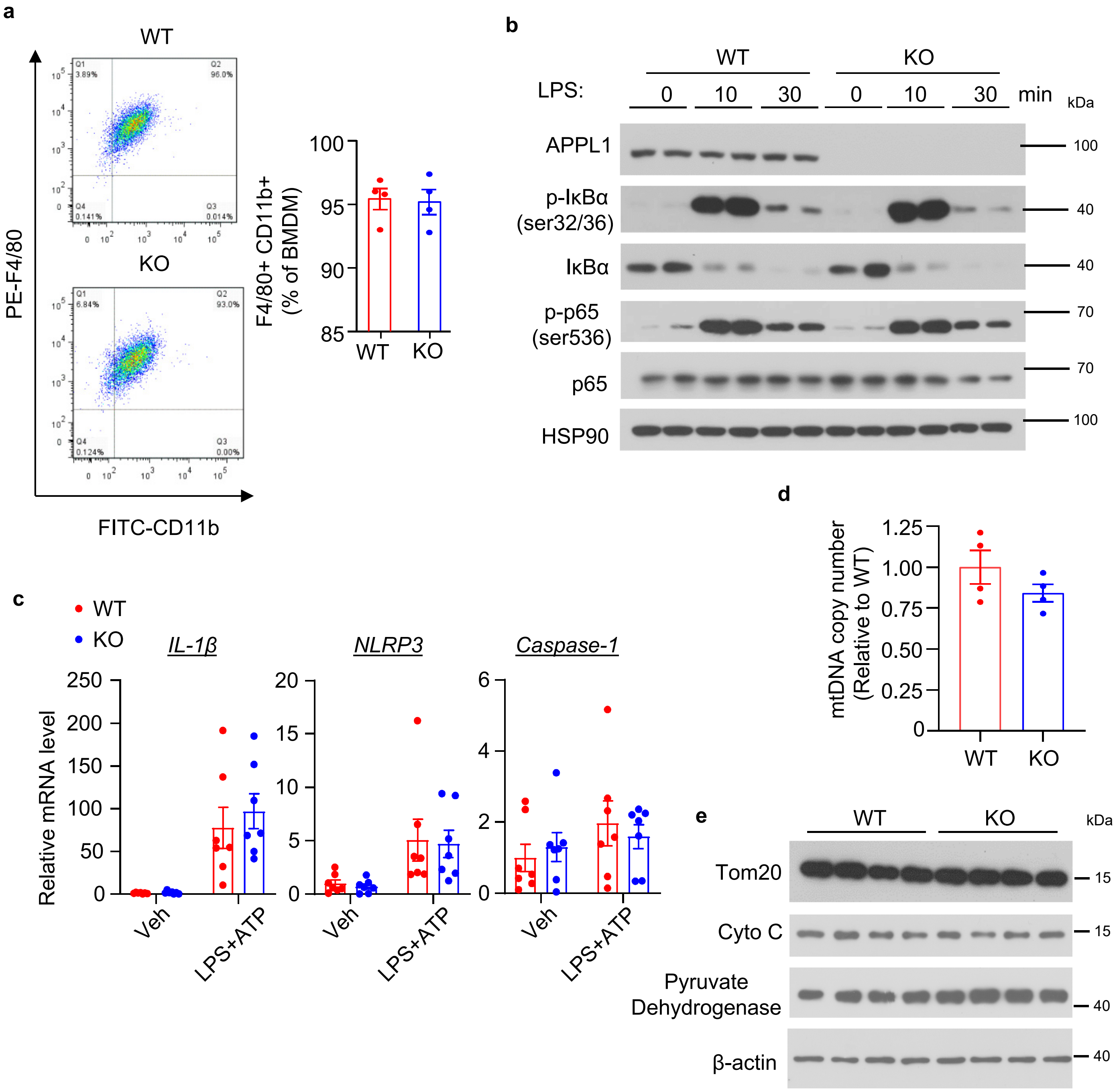

Supplementary figure 2. Effect of APPL1 deficiency on macrophage differentiation, NF-kB pathway and mitochondrial mass. (a-e) BMDM from 10-12-week-old APPL1 KO mice and their WT controls were used. (a) Bone marrow cells from APPL1 KO mice and their WT controls were differentiated into BMDM using L929 conditioned medium for 7 days. The cells were then stained with an anti-F4/80 antibody conjugated with PE fluorophore and an anti-CD11b antibody conjugated with FITC fluorophore, followed by flow cytometry analysis. n=4 biologically independent samples. (b) The BMDM were stimulated with LPS (100 ng/ml) for indicated time, followed by immunoblotting analysis of APPL1, total p65 and its phosphorylation at serine 536, total IκBα and its phosphorylations at serine 32 and 36 and β-actin as loading control. (c) QPCR analysis of mRNA expression of the genes related to inflammasome in the BMDM, and the data was normalized with *GAPDH*. n=7 biologically independent samples. (d) QPCR of mtDNA copy number by measuring d-loop region of mtDNA, and the data was normalized with nuclear DNA Tert. n=4 biologically independent samples. (e) The BMDM were treated with LPS (100 ng/ml) for 20 hours, followed by immunoblotting analysis of the mitochondrial proteins. n=4 biologically independent samples. Representative immunoblotting images and flow cytometry plot are shown. Data are displayed as mean ± SEM. Statistical significance was tested using two-tailed student's *t*-test (a, c & d).

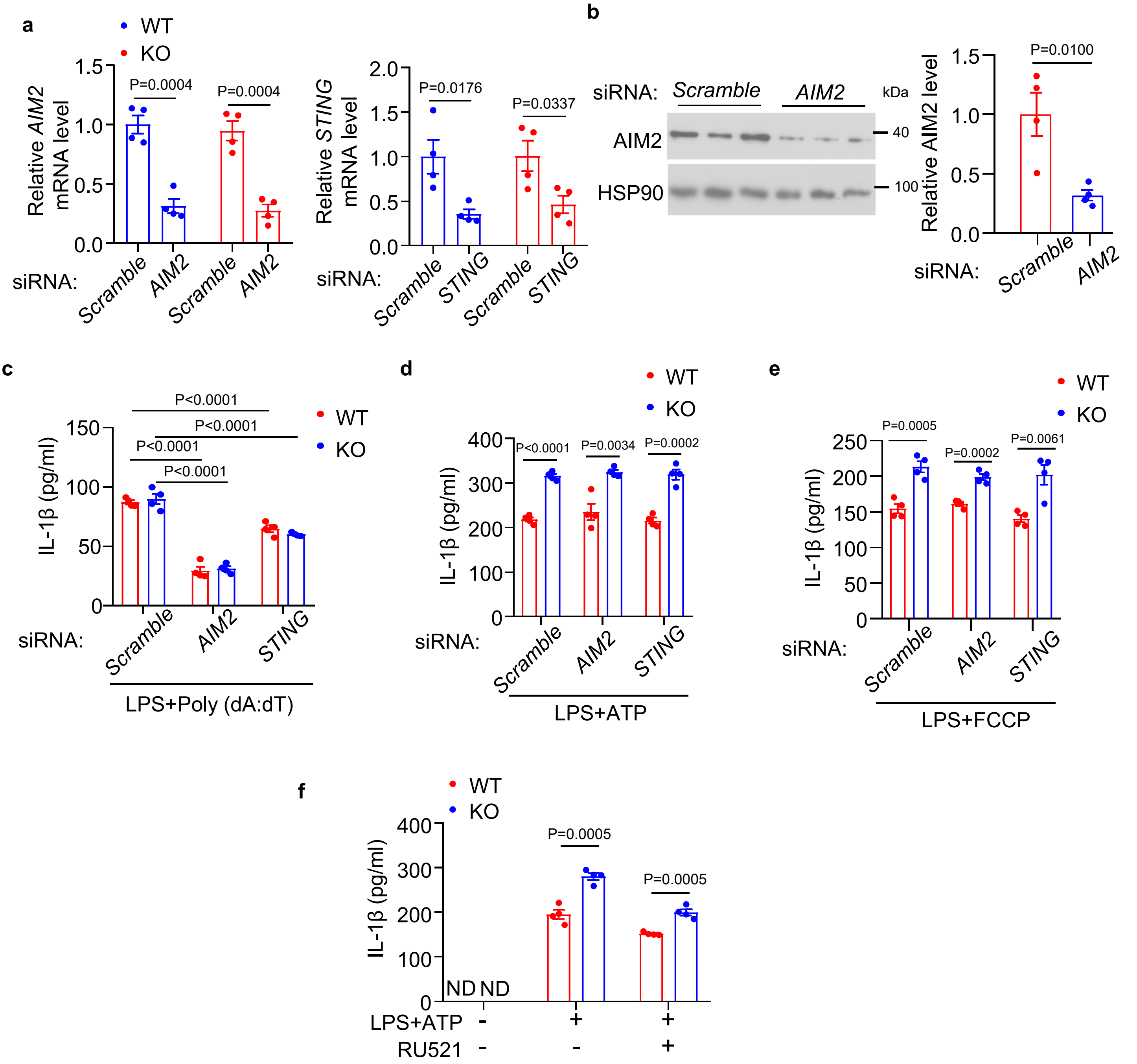

Supplementary figure 3. Effect of siRNA-mediated silencing of AIM2 or STING or pharmacological inhibition of cCAS on APPL1 deficiency-induced IL-1β oversecretion in BMDM. BMDM from APPL1 KO mice and their WT littermates transfected with siRNA targeting *AIM2* and *STING* or *Scramble* using DharmaFECT 3 transfection reagent for 24 hours, followed by LPS priming for 20 hours and stimulation with ATP, Poly (dA:dT) or FCCP as indicated. (a) qPCR analysis of *AIM2* and *STING* level normalized with *GAPDH* after the transfection. n=4 biologically independent samples. (b) Immunoblotting analysis of AIM2 and HSP90 after the transfection. The bar chart is densitometry analysis of relative AIM2 normalized with HSP90. n=4 biologically independent samples. (c-e) The transfected and LPS-primed BMDM were stimulated with Poly (dA:dT) (Panel c), ATP (Panel d) or FCCP (Panel e) for 6 hours, 4 hours or 6 hours, respectively. (f) Before the ATP stimulation, LPS-primed BMDM were pre-treated with RU521 for 4 hours. (c-f) IL-1β levels in the cell culture supernatant were measured by the ELISA. n=4 biologically independent samples. Data are displayed as mean ± SEM. Statistical significance was tested using two-tailed student's *t*-test (a, b, d, e & f) or one-way ANOVA with post hoc Bonferroni correction (c).

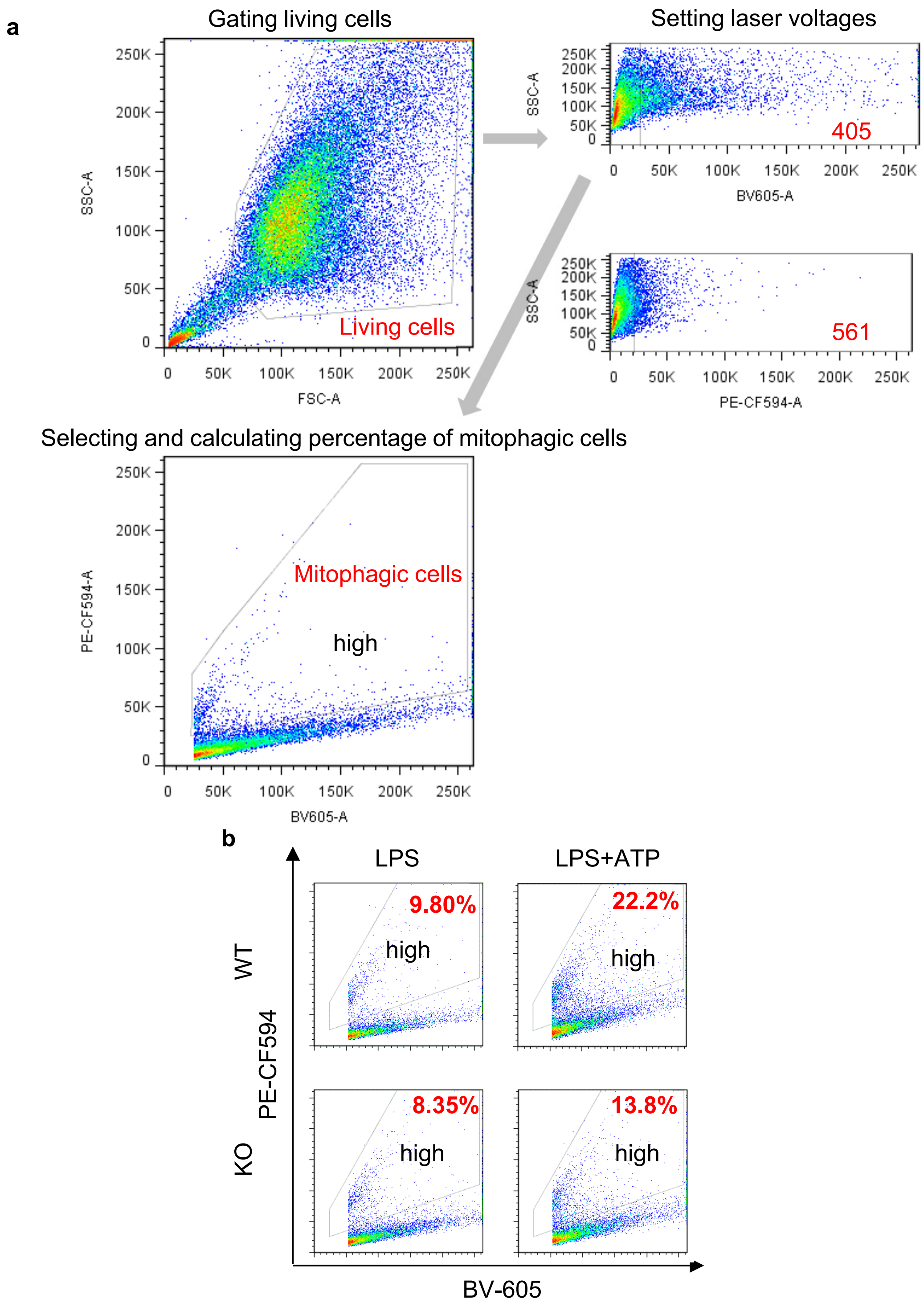

Supplementary figure 4. Flow cytometry analysis of mitophagy using Mito-Keima. The BMDM were infected with adenovirus encoding mitochondrial target-Keima (Mito-Keima) for 24 hours, followed by priming with LPS for 20 hours and ATP stimulation for 30 minutes. The cells subjected to flow cytometry analysis using BV605 and PE-CF594 detector. The cells with high ratio of PE-CF594/BV605 are defined as mitophagic cells and quantified. (a) The gating strategy for identification of mitophagic cells. (b) Representative flow cytometry plot of Figure 5b.

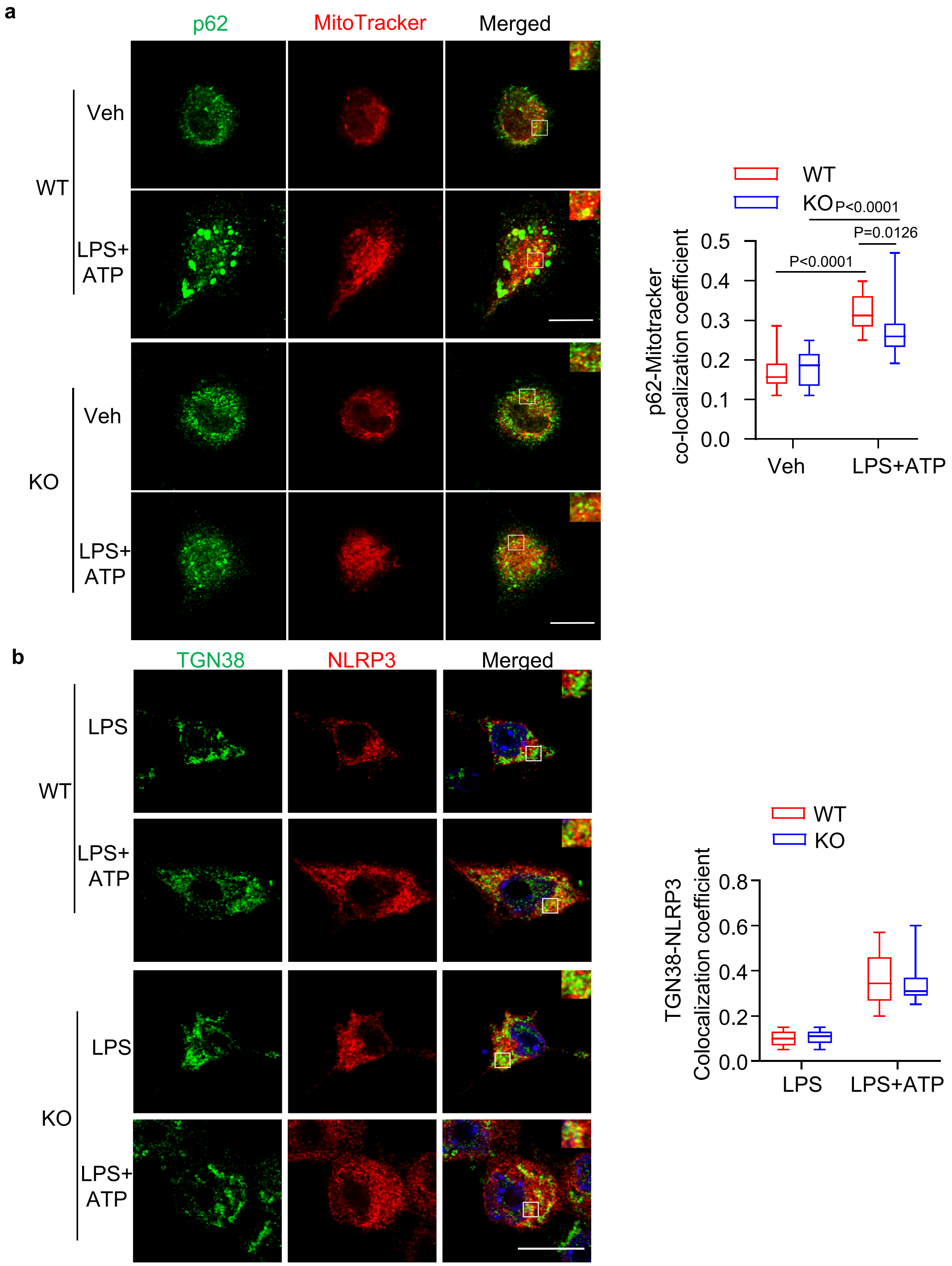

Supplementary figure 5. APPL1 deficiency abrogates p62 recruitment to the mitochondria but has no obvious effect on NLRP3 recruitment to dispersed trans-Golgi under LPS+ATP stimulation condition. BMDM from APPL1 KO mice and their WT controls were primed with LPS for 20 hours, followed by stimulation with ATP for 60 min in panel A and 10 min in panel B. (a) p62 (green) and MitoTracker Deep Red (Red) as indicated. Scale bar: 10 µm. The right panel is the co-localization coefficient between $p62^+$ and $Mitotracker^+$ puncta. n=50 biologically independent cells. (b) Immunofluorescence staining of TGN38 (Green) and NLRP3 (Red) in the BMDM. Scale bar: 10 µm. The right panel is the co-localization coefficient between $TGN38^+$ and $NLRP3^+$ puncta. n=50 biologically independent cells. Representative images are shown. *p<0.05 and ***p<0.001. Data are displayed as mean ± SEM. In box and whisker plots, the whiskers extend to the minimum and maximum values, while the box presents the 25th and 75th percentiles with the central line at the median. Statistical significance was tested using Kruskal-Wallis test with Dunn's test (a & b).

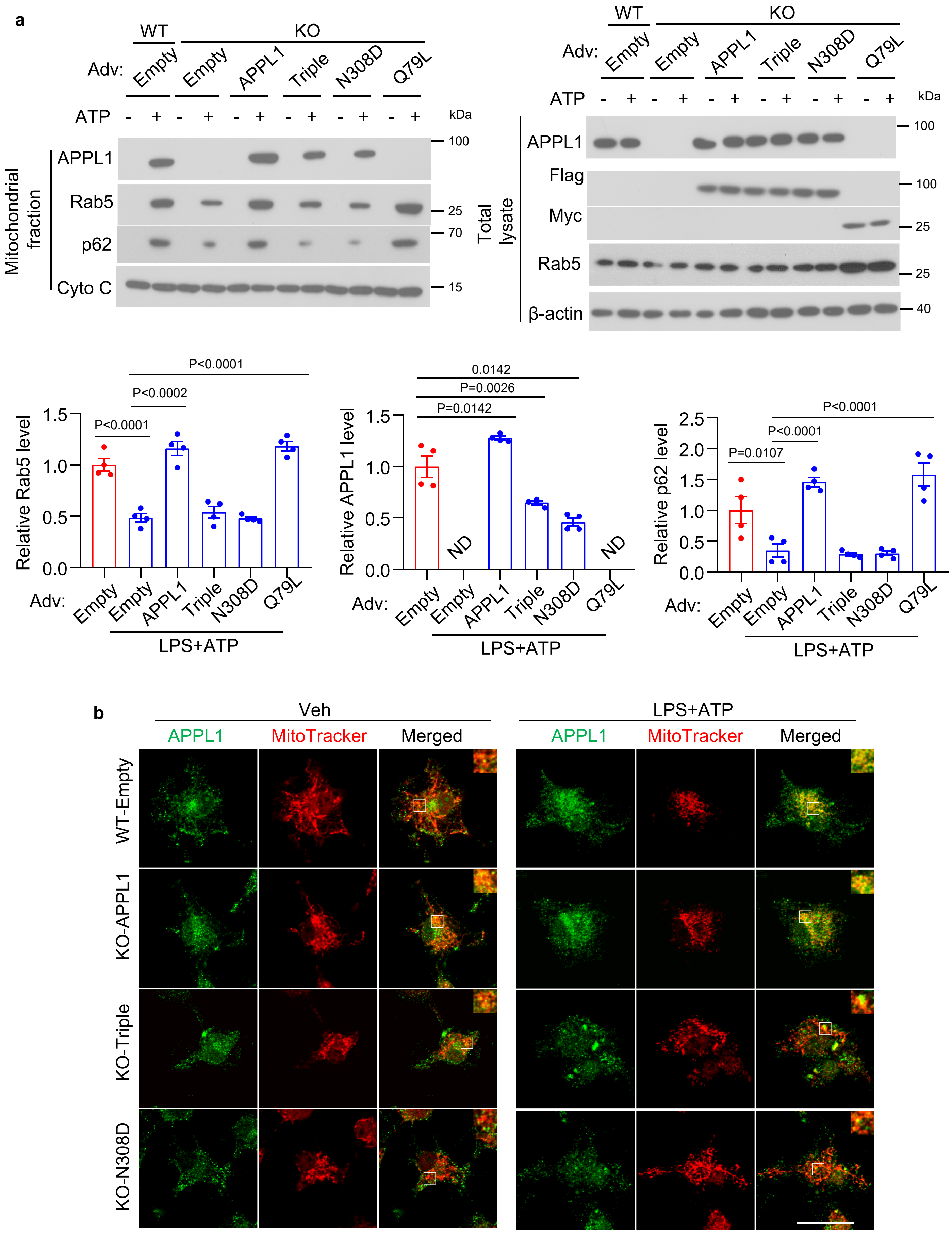
a
WT
KO
Adv:
Empty
Empty
APPL1
Triple
N308D
Q79L
ATP
kDa
Mitochondrial fraction
APPL1
Rab5
p62
Cyto C
100
25
70
15
Total lysate
APPL1
Flag
Myc
Rab5
β-actin
100
100
25
25
40
Relative Rab5 level
P<0.0001
P<0.0002
P<0.0001
LPS+ATP
Relative APPL1 level
0.0142
P=0.0026
P=0.0142
ND
ND
Relative p62 level
P<0.0001
P<0.0001
P=0.0107
b
Veh
LPS+ATP
APPL1
MitoTracker
Merged
WT-Empty
KO-APPL1
KO-Triple
KO-N308D

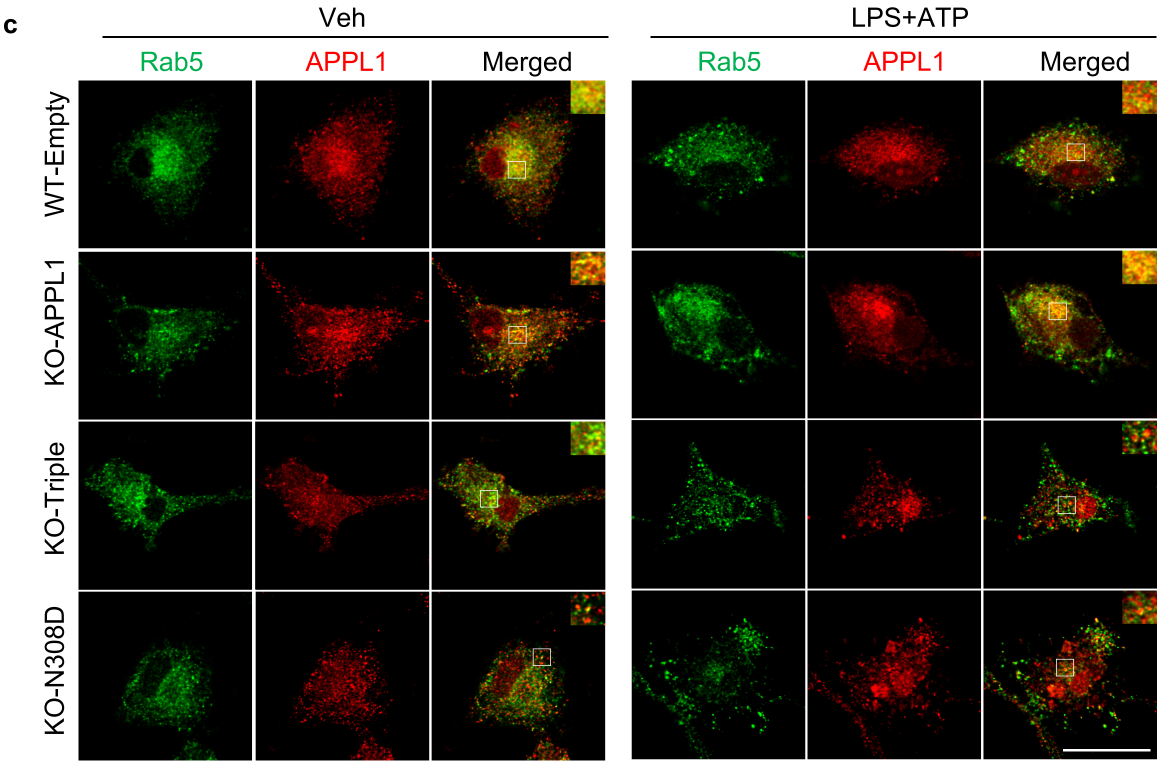

Supplementary figure 6. The Rab5 binding and endosomal localization ability of APPL1 is required for the recruitment of APPL1 and Rab5 to the mitochondria upon NLRP3 agonist stimulation. BMDM were isolated from APPL1 KO mice and their WT controls, followed by infection with the recombinant adenovirus encoding wild-type APPL1 (APPL1), APPL1 triple mutants (Triple), APPL1 N308D mutant (N308D) or empty adenovirus as control for 24 hours. The infected cells were primed with LPS for 20 hours and then incubated with MitoTracker Deep Red (Panel b only), followed by ATP stimulation for 15 min in Panel a and 1 hour in Panel b and c. BMDM without any stimulation is labelled as "Veh". (a) The stimulated cells were subjected to mitochondrial fractionation, followed by immunoblotting analysis as indicated. The bar charts at the lower panels are relative abundance of APPL1, Rab5 and p62 normalized with Cyto C in the mitochondrial fraction. n=4 biologically independent samples. The samples were collected from the same experiment and blots were processed in parallel. (b) Immunofluorescence staining of APPL1 (Green) and MitoTracker Deep red (Red) in the BMDM. (c) Immunofluorescence staining of Rab5 (Green) and APPL1 (Red) in the BMDM. Scale bar: 10 µm. Representative images are shown. Data are displayed as mean ± SEM. Statistical significance was tested using one-way ANOVA with post hoc Bonferroni correction (a).

a

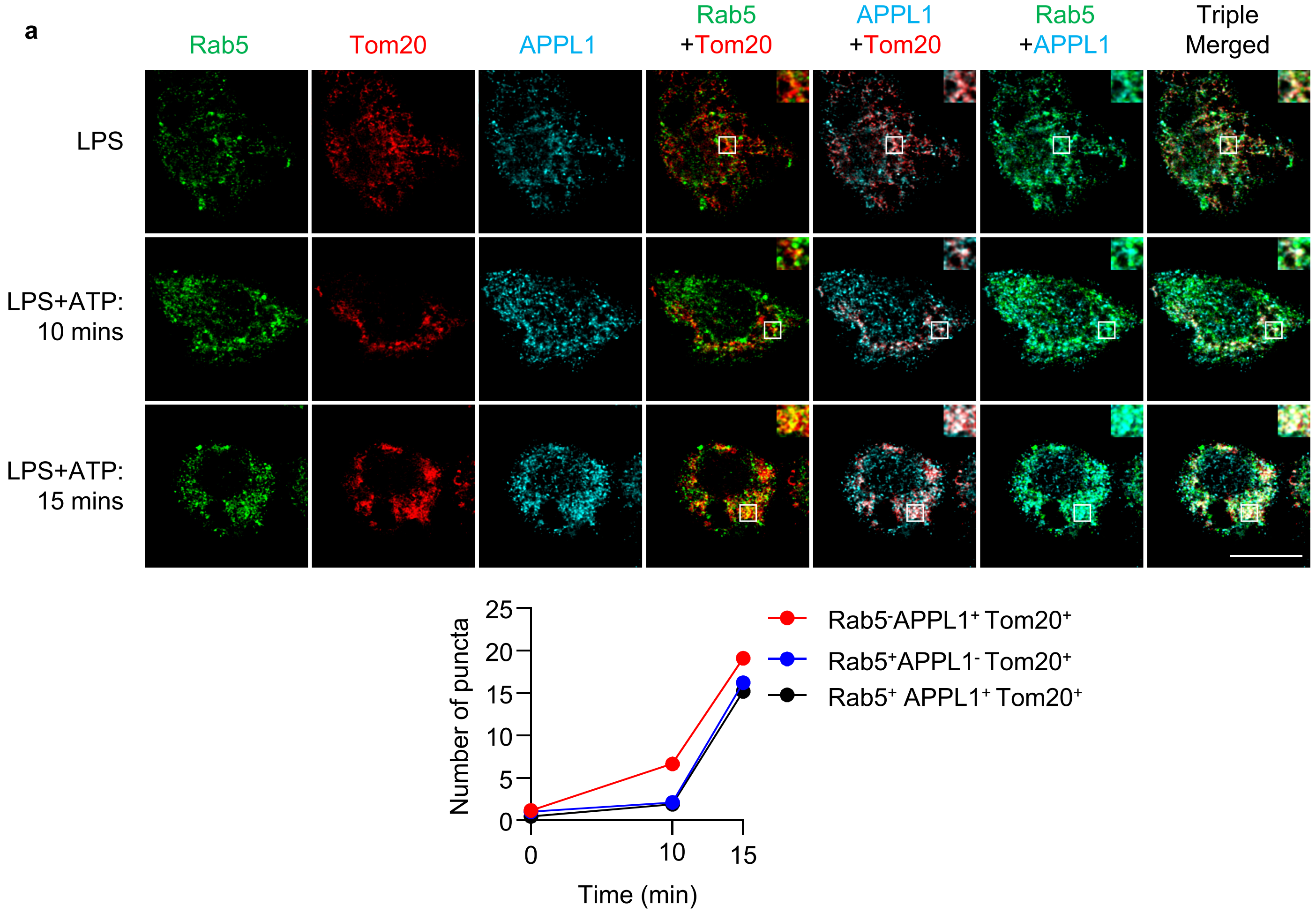
Rab5
Tom20
APPL1
Rab5 +Tom20
APPL1 +Tom20
Rab5 +APPL1
Triple Merged
LPS
LPS+ATP: 10 mins
LPS+ATP: 15 mins
Number of puncta
Time (min)
Rab5$^-$APPL1$^+$ Tom20$^+$
Rab5$^+$APPL1$^-$ Tom20$^+$
Rab5$^+$ APPL1$^+$ Tom20$^+$

b

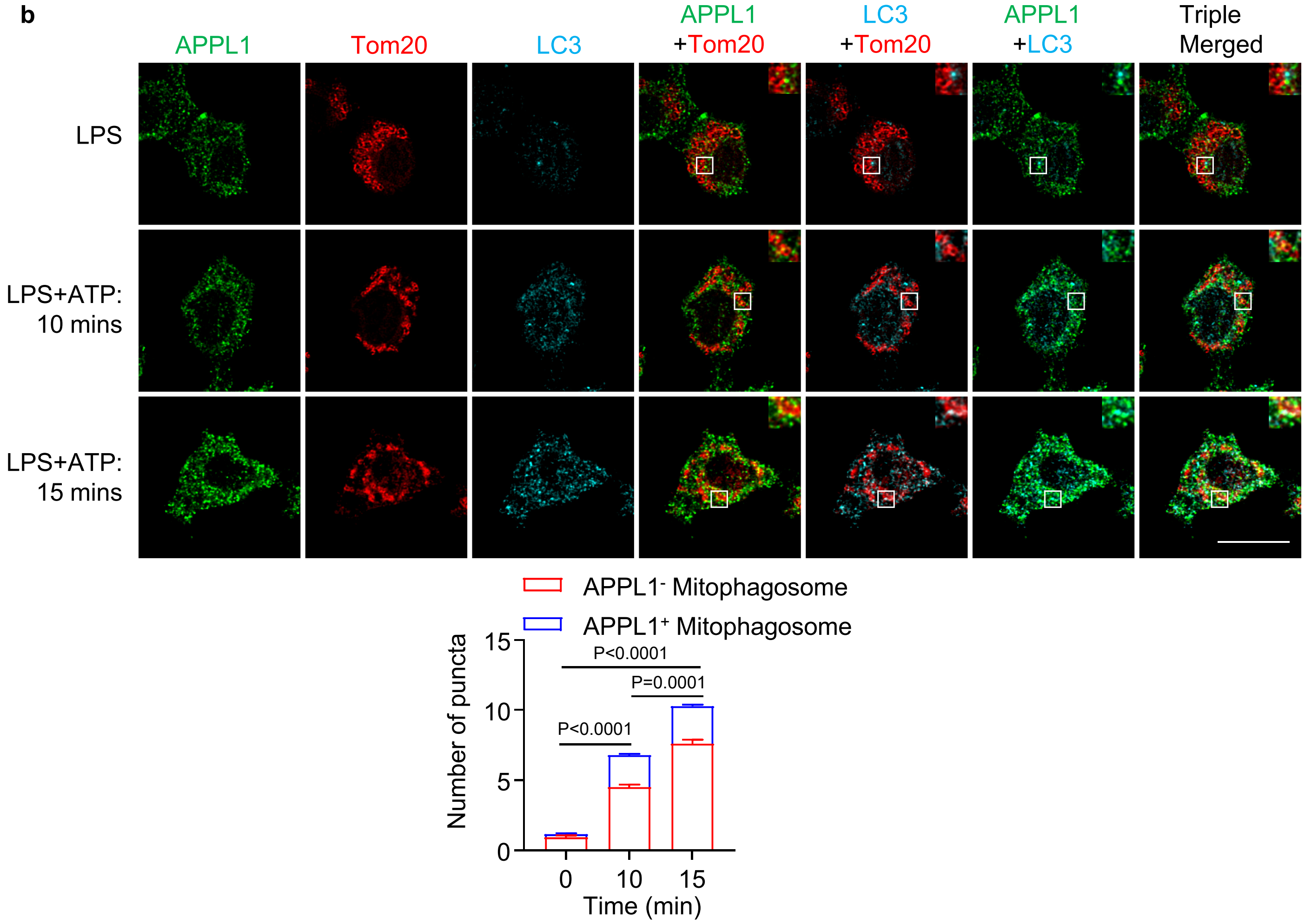
APPL1
Tom20
LC3
APPL1 +Tom20
LC3 +Tom20
APPL1 +LC3
Triple Merged
LPS
LPS+ATP: 10 mins
LPS+ATP: 15 mins
APPL1$^-$ Mitophagosome
APPL1$^+$ Mitophagosome
Number of puncta
P<0.0001
P=0.0001
P<0.0001
Time (min)

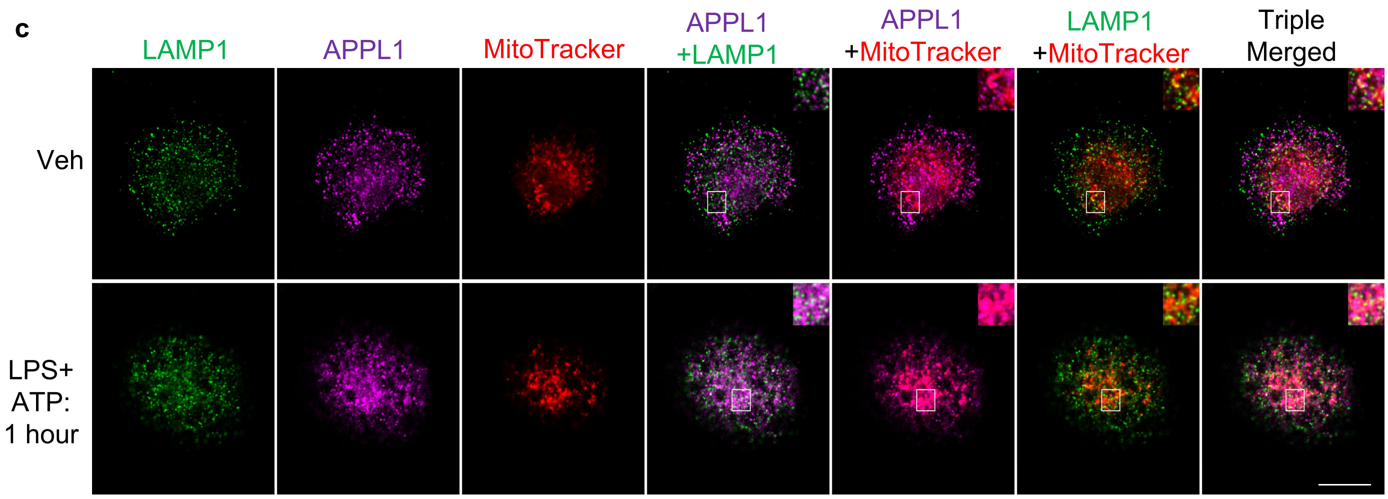

Supplementary figure 7. Effect of NLRP3 inflammasome activation on intracellular localization of APPL1. BMDM from C57BL/6J mice were primed with or without LPS, followed by treatment with ATP as indicated time points. The BMDM were incubated with MitoTracker Deep Red (Red) for 30 minutes before immunofluorescence staining. (a) Immunofluorescence staining of Rab5 (Green), Tom20 (Red) and APPL1 (Cyan) in the BMDM. (b) Immunofluorescence staining of APPL1 (Green), LC3 (Cyan) and Tom20 (Red) in the BMDM. (c) BMDM without any stimulation is labelled as "Veh". Immunofluorescence staining of APPL (purple) and LAMP1 (green) and MitoTracker Deep red (Red). Scale bar: 10 µm. Representative images are shown. Statistical significance was tested using Kruskal-Wallis test with Dunn's test (b).

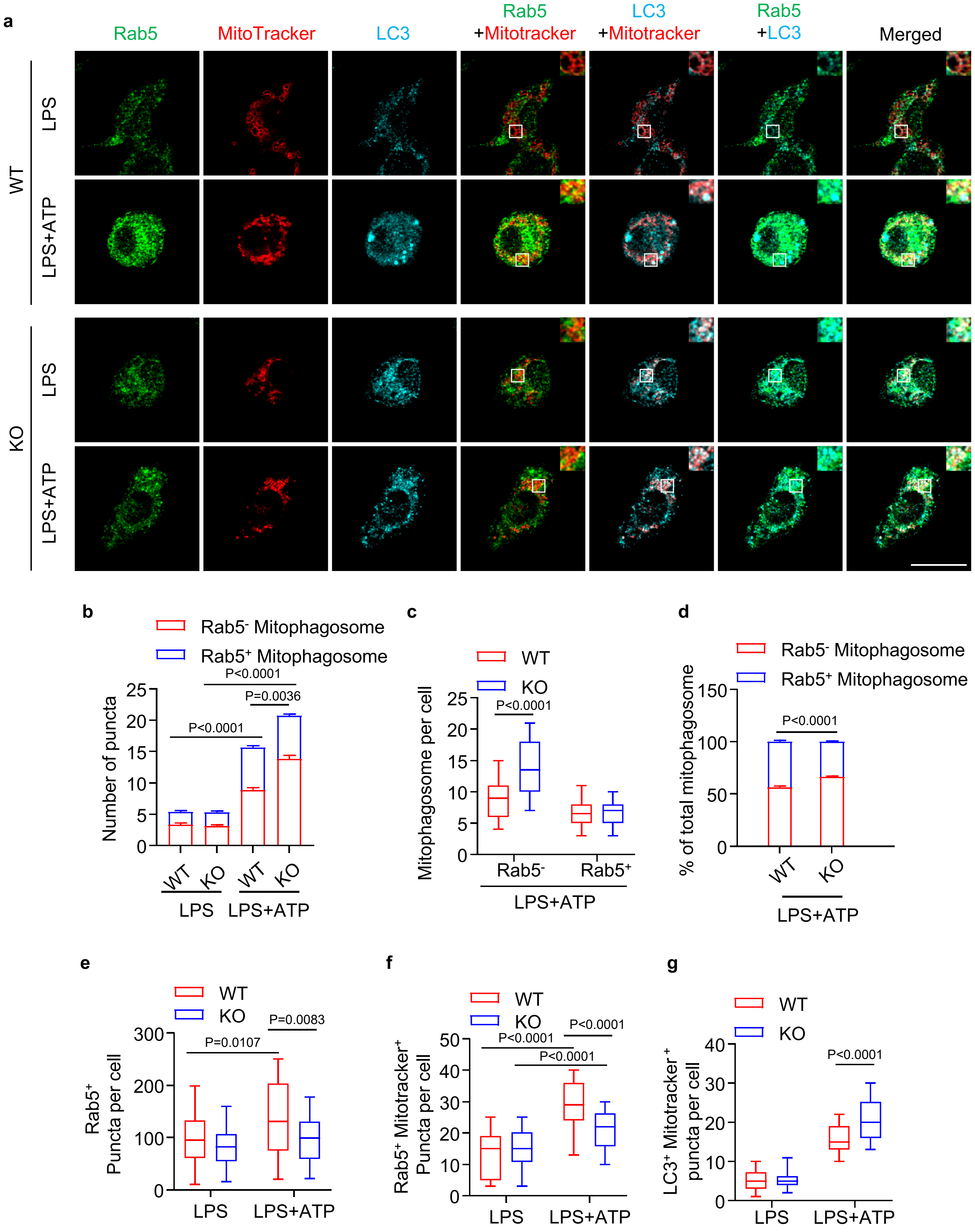

a
Rab5
MitoTracker
LC3
Rab5 +Mitotracker
LC3 +Mitotracker
Rab5 +LC3
Merged
WT
KO
LPS
LPS+ATP
b
Rab5- Mitophagosome
Rab5+ Mitophagosome
Number of puncta
P<0.0001
P=0.0036
P<0.0001
WT KO WT KO
LPS LPS+ATP
c
WT
KO
Mitophagosome per cell
P<0.0001
Rab5- Rab5+
LPS+ATP
d
Rab5- Mitophagosome
Rab5+ Mitophagosome
% of total mitophagosome
P<0.0001
WT KO
LPS+ATP
e
WT
KO
Rab5+ Puncta per cell
P=0.0107
P=0.0083
LPS LPS+ATP
f
WT
KO
Rab5+ Mitotracker+ Puncta per cell
P<0.0001
P<0.0001
P<0.0001
LPS LPS+ATP
g
WT
KO
LC3+ Mitotracker+ puncta per cell
P<0.0001
LPS LPS+ATP

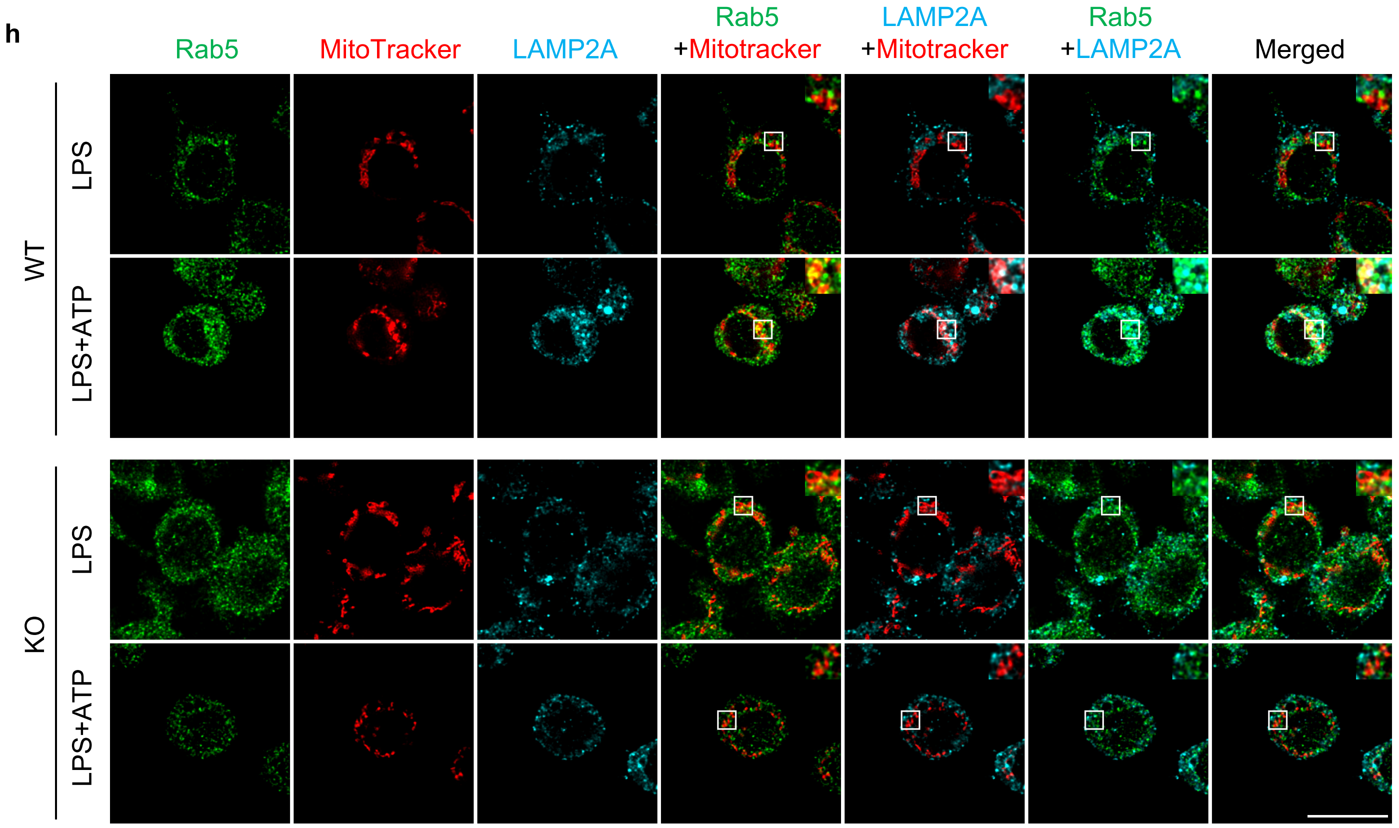

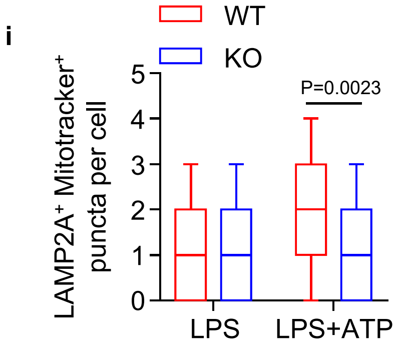

Supplementary figure 8. Effect of APPL1 ablation on the recruitment of Rab5+ endosomes to mitophagosomes, and formation of mitophagosome and mitochondrial autolysosomes. BMDM were isolated from 10-12 weeks old APPL1 KO mice and their WT controls, followed by priming with LPS for 20 hours. The LPS-primed BMDM were stained with Mitotracker Deep Red for 30 minutes, followed by treatment with ATP for 30 minutes in Panel a or 60 min in Panel h. (a) Immunofluorescence staining of Rab5 (Green), LC3 (Cyan) and Mitotracker Deep red (Red) in the BMDM. (b-g) Quantification of number or percentage of $Rab5^+$ or $Rab5^-$ mitophagosomes, $Rab5^+$ puncta, $Rab5^+$ mitochondria as indicated. (h) Immunofluorescence staining of Rab5 (Green), LAMP2A (Cyan) and Mitotracker Deep red (Red) in the BMDM. (i) Quantification of $LAMP2A^+$ mitochondria. Of note, a very rare mitochondrial autolysosome are $Rab5^+$. n=50 biologically independent cells. Scale bar: 100 µm. Representative images are shown. In box and whisker plots, the whiskers extend to the minimum and maximum values, while the box presents the 25th and 75th percentiles with the central line at the median. Statistical significance was tested using two-tailed student's *t*-test (d) or Mann-Whitney U test (c, d, g & i), one-way ANOVA with post hoc Bonferroni correction (f) or Kruskal-Wallis test with Dunn's test (b & e).

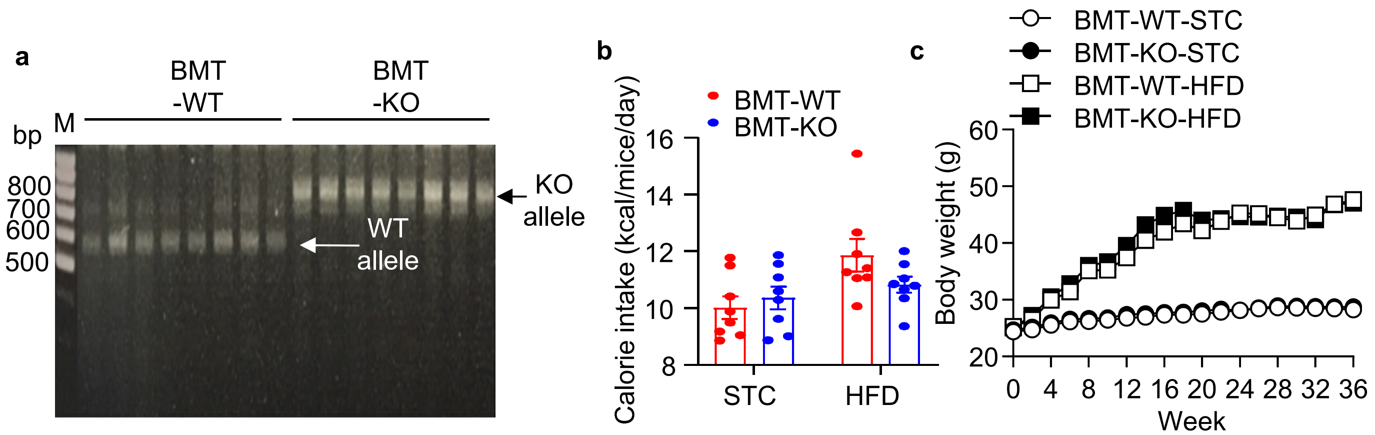

Supplementary figure 9. Generation of hematopoietic-specific APPL1 knockout mice and their daily calorie intake and body weight. (a) Peripheral blood was collected BMT-KO mice and BMT-WT controls, followed by genomic DNA extraction. DNA was used for PCR genotyping with primers targeting APPL1 KO allele and WT allele as indicated. M: 100 bp DNA ladder. (b) Daily calorie intake. n=8. (c) Body weight of BMT-KO mice and BMT-WT mice during dietary intervention. The week of bone marrow transplantation is defined as week 0. BMT-WT-STC: n=7; BMT-KO-STC: n=8; BMT-WT-HFD: n=12; BMT-KO-HFD: n=8 biologically independent animals. Data are displayed as mean ± SEM.

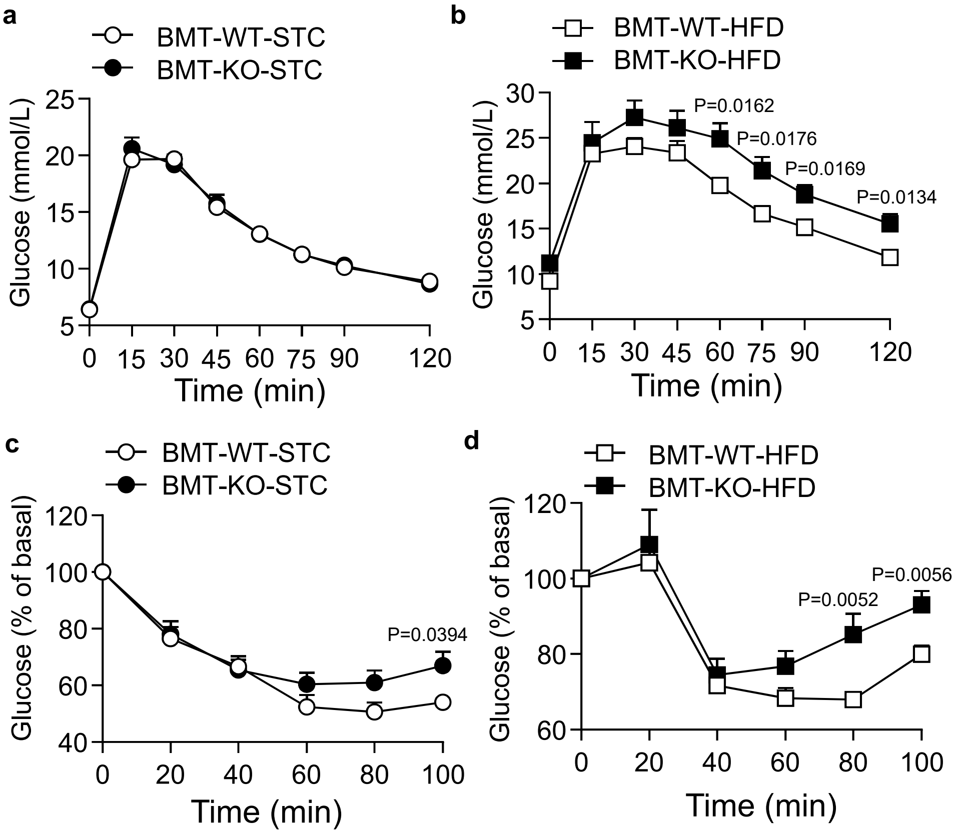

Supplementary figure 10. Hematopoietic deficiency of APPL1 exacerbates dietary-induced glucose dysregulation in mice. 44-week-old male BMT-APPL1 KO mice and BMT-WT mice fed with STC or HFD were used. (a-b) Glucose tolerance test. 2 g/kg and 1.25 g/kg glucose was intraperitoneally injected into the 16-hour fasted mice fed with STC and HFD, respectively. BMT-WT-STC: n=7; BMT-KO-STC: n=8 biologically independent animals (c-d) Insulin tolerance test. 0.75 IU/kg and 1.25 IU/kg insulin was intraperitoneally injected into the 6-hour fasted mice fed with STC and HFD, respectively. BMT-WT-HFD: n=8; BMT-WT-HFD: n=8 biologically independent animals. Data are displayed as mean ± SEM. Statistical significance was tested using two-tailed student's *t*-test (a-d).

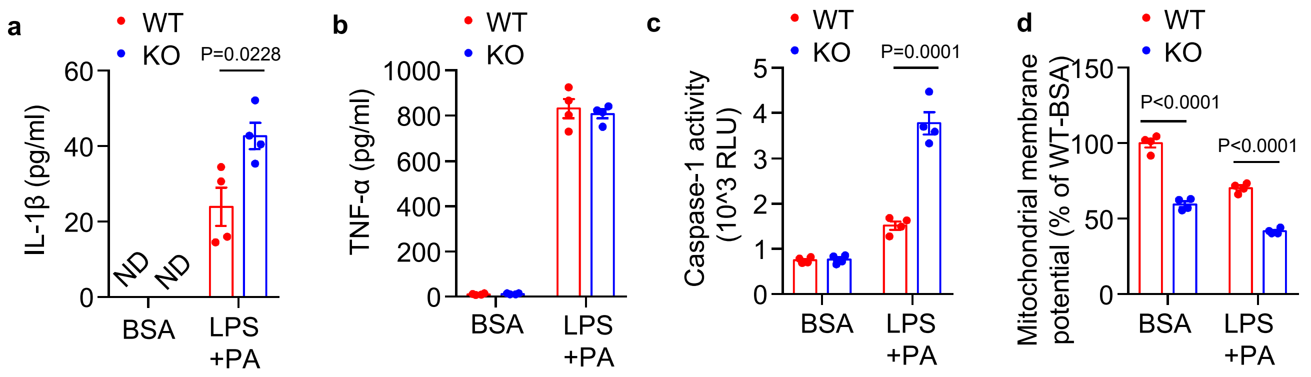

Supplementary figure 11. APPL1 deficiency promotes palmitic acid-induced NLRP3 inflammasome activation and mitochondrial dysfunction. BMDM from APPL1 KO mice and their WT controls were primed with LPS (100 ng/ml) for 20 hours, followed by stimulation with palmitic acid (PA; 0.5 mM) for 24 hours. (a) IL-1β, (b) TNF-α and (c) caspase-1 activity in the cell culture supernatant were measured as described in Figure 1. n=4 biologically independent samples. (d) Mitochondrial membrane potential in BMDM measured by TMRE assay. The data is presented as percentage of WT-BSA. n=4 biologically independent samples. Undetectable (ND). Data are displayed as mean ± SEM. Statistical significance was tested using two-tailed student's *t*-test (a-d).

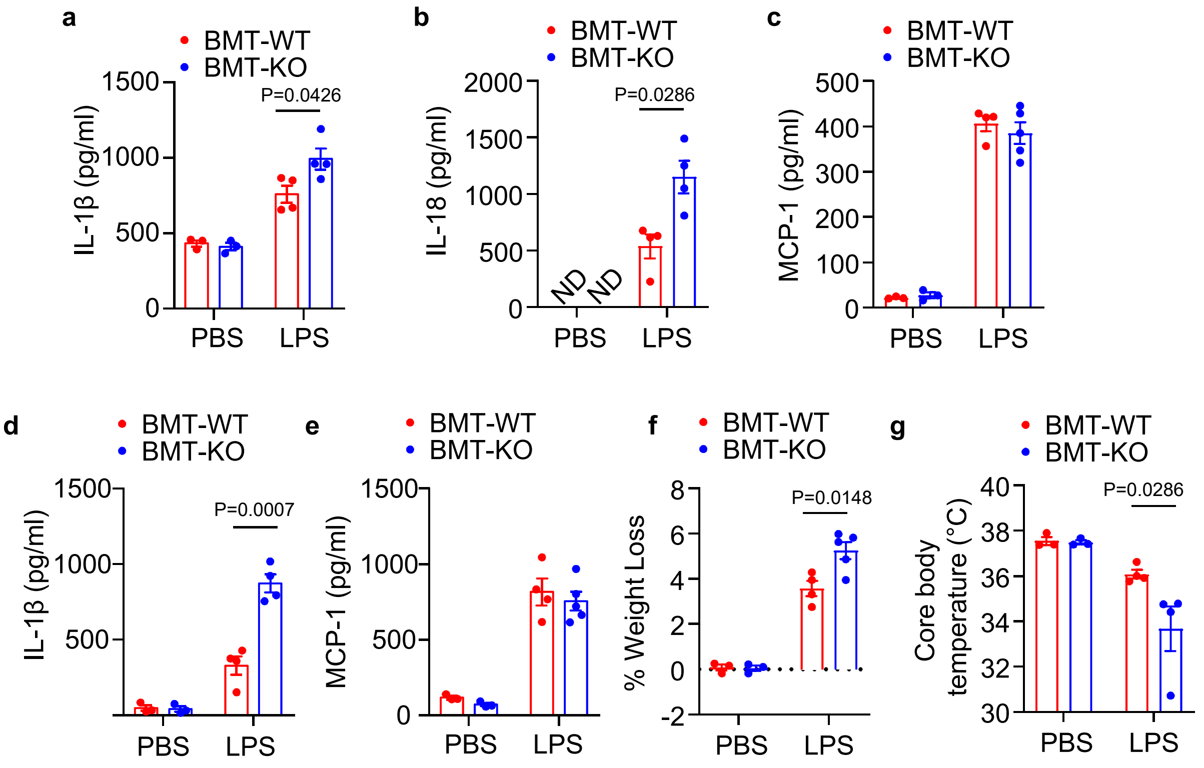

Supplementary figure 12. Hematopoietic-specific APPL1 ablation aggravates LPS-induced sepsis and IL-1β production in mice. BMT-APPL1 KO mice and BMT-WT controls were subjected to intraperitoneal injection of LPS at a dosage of 10 mg/kg or PBS as control. The mice were sacrificed after 6 hours. Circulating (a) IL-1β, (b) IL-18 and (c) MCP-1 level in serum of mice after injection with LPS or PBS. Peritoneal lavage fluid were collected for measurement of (d) IL-1β and (e) MCP-1. (f) Weight loss and (g) core body temperature of the mice after injection with LPS or PBS. (a, b, d & g) BMT-WT-PBS & BMT-KO-PBS: n=3; BMT-WT-LPS & BMT-KO-LPS: n=4 biologically independent animals. (c, e & f) BMT-WT-PBS & BMT-KO-PBS: n=3; BMT-WT-LPS: n=4; BMT-KO-LPS: n=5 biologically independent animals. Data are displayed as mean ± SEM. Statistical significance was tested using two-tailed student's *t*-test (a, c, d, e & f) or Mann-Whitney U test (b & g).

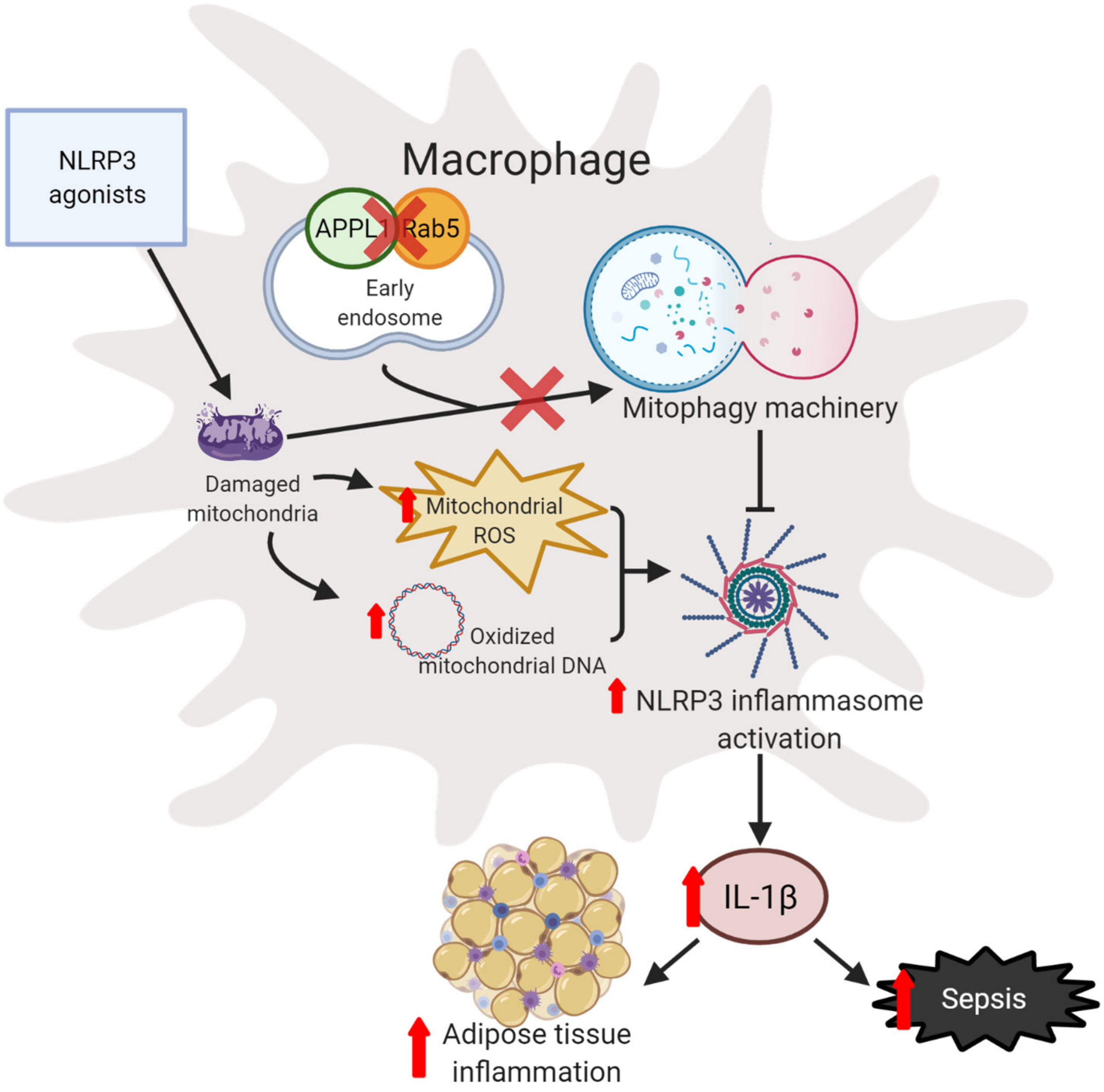

Supplementary figure 13. Endosomal APPL1-mediated mitophagy restricts NLRP3 inflammasome activation via interaction with Rab5 in macrophages. APPL1 interacts with Rab5 on the early endosome to facilitate mitophagy, which in turn removes danger mitochondrial signals, thereby restricting NLRP3 inflammasome activation in macrophages. APPL1 deficiency in hematopoietic exacerbates IL-1β production in obesity and endotoxin-induced sepsis mouse models. Abbreviations: ROS (Reactive oxygen species). Figure created with BioRender.com.

	BMT-WT -STC	BMT-KO -STC	BMT-WT -HFD	BMT-KO -HFD
**Insulin (µg/L)**	0.323±0.033	0.470±0.035 (P=0.0127)	0.965±0.166	1.727±0.158 (P=0.0111)
**Glucose (mmol/L)**	6.386±0.373	6.463±0.251	8.938±0.293	11.2±0.731 (P=0.0123)
**HOMA-IR**	2.578±0.2	3.887±0.323* (P=0.0055)	11.407±2.025	20.012±1.947 (P=0.0098)
**AST (U/L)**	-	-	135.785±7.552	164.434±9.171 (P=0.0328)
**ALT (U/L)**	-	-	58.768±4.114	57.392±3.996
**Adiponectin (µg/ml)**	18.024±0.773	18.157±0.633	14.758±1.492	11.292±1.01
**MCP1 (pg/ml)**	-	-	115.963±7.751	149.044±12.79 (P=0.0315)
**FFA (mM)**	0.355±0.014	0.405±0.044	0.807±0.033	0.883±0.051

Supplementary Table 1. Metabolic profile of BMT-APPL1 KO mice and BMT-WT mice. APPL1-BMT-APPL1 KO mice and BMT-WT controls were fed with STC or HFD for 36 weeks. Insulin, blood glucose, AST, ALT and adiponectin were measured in serum collected from mice after 6 hours of fasting. MCP-1 and FFA were measured in serum collected after 16 hours of fasting. BMT-WT-STC: n=3; BMT-KO-STC: n=3; BMT-WT-HFD: n=7; BMT-KO-HFD; n=8 biologically independent animals. Data are displayed as mean ± SEM. Statistical significance was tested using two-tailed student's *t*-test (Glucose, HOMA-IR, AST, Adiponectin, MCP-1 and FFA) or Mann-Whitney test (Insulin & ALT) .

Gene	Forward (5'-3')	Reverse (5'-3')
Mitochondrial CO1	GCCCCAGATATAGCATTCCC	GTTCATCCTGTTCCTGCTCC
18S rRNA	TAGAGGGACAAGTGGCGTTC	CGCTGAGCCAGTCAGTGT
Mitochondrial D-loop	AATCTACCATCCTCCGTGAAACC	TCAGTTTAGCTACCCCCAAGTTTAA
Nuclear DNA Tert	CTAGCT CATGTGTCAAGACCCTCTT	GCCAGCACGTTTCTCTCGTT
IL-1β	CTGGTGTGTGACGTTCCCATTA	CCGACAGCACGAGGCTTT
NLRP3	TGCTCTTCACTGCTATCAAGCCCT	ACAAGCCTTTGCTCCAGACCCTAT
Caspase-1	ACAAGGCACGGGACCTATG	TCCCAGTCAGTCCTGGAAATG
GAPDH	CTCATGACCACAGTCCATGC	CACATTGGGGGTAGGAACAC
AIM2	GATTCAAAGTGCAGGTGCGG	TCTGAGGCTTAGCTTGAGGAC
STING	AAATAACTGCCGCCTCATTG	ACAGTACGGAGGGAGGAGG

Supplementary Table 2. Primer sequences used for QPCR analysis in this study

Antibody	Catalog number and Brand	Usage and Dilution factor
HSP90	#7874, Cell Signaling Technology	WB (1:2500)
NLRP3	#15101, Cell Signaling Technology	WB (1:2500)
NLRP3	#ab270449, Abcam	IF (1:100)
Caspase-1	#AG-20B-0042-C100, Adipogen	WB (1:2500), IF (1:100)
Cleaved IL-1β	#63124, Cell Signaling Technology	WB (1:1000)
IL-1β	#12242, Cell Signaling Technology	WB (1:2000)
Cleaved caspase-1	#67314, Cell Signaling Technology	WB (1:2500)
Tom20	#42406, Cell Signaling Technology	WB (1:2500), IF (1:200)
Tom20	#sc-17764, Santa Cruz	IF (1:50)
β-actin	#sc-47778, Santa Cruz	WB (1:5000)
LC3B	#12741, Cell Signaling Technology	WB (1:2500), IF (1:100)
Rab5	#46449, Cell Signaling Technology	IF (1:400)
APPL1	#3858, Cell Signaling Technology	WB (1:2500), IF (1:200)
APPL1	#sc-271909, Santa Cruz	IF (1:50)
F4/80	#MCA497RT, Bio-Rad	IF (1:100)
IκBα	#9242, Cell Signaling Technology	WB (1:2500)
p-IκBα (ser32/36)	#9241, Cell Signaling Technology	WB (1:2500)
p65	#8242, Cell Signaling Technology	WB (1:2500)
p-p65 (ser536)	#3033, Cell Signaling Technology	WB (1:2500)
LAMP1	#sc-20011, Santa Cruz	IF (1:100)
LAMP2A	#ab18528, Abcam	IF (1:100)
p62	#5114, Cell Signaling Technology	IF (1:100)
Cyto C	#4272, Cell Signaling Technology	WB (1:2000)
AIM2	#sc-293174, Santa Cruz	WB (1:2500)
Alexa Fluor® 647 Anti-TOMM20	#ab205487, Abcam	IF (1:100)
TGN38	#sc-166594, Santa Cruz	IF (1:50)
PE-F4/80	# 123110, BioLegend	FC (1:100)
FITC-Cd11b	# 101206, BioLegend	FC (1:100)

Supplementary Table 3. Antibodies used in this study. Western blot (WB); Immunofluorescence staining (IF); Flow cytometry (FC).

Gene	Forward (5'-3')	Reverse (5'-3')
AIM2	ACAUAGACACUGAGGGUAU	AUACCCUCAGUGUCUAUGU
STING	CGAAATAACTGCCGCCTCA	UGAGGCGGCAGUUAUUUCG
Scramble	UUCUCCGAACGUGUCACGUTT	ACGUGACACGUUCGGAGAATT

Supplementary Table 4. Sequences of siRNA used in this study
