# Supplementary material for: The APPL1-Rab5 axis restricts NLRP3 inflammasome activation through early endosomal-dependent mitophagy in macrophages
Source: Nat Commun. 2021 Nov 17;12:6637. doi: 10.1038/s41467-021-26987-1 (PMC8599493; doi:10.1038/s41467-021-26987-1)
Supplement: Supplementary file 3 — Reporting Summary [file 41467_2021_26987_MOESM3_ESM.pdf]

nature portfolio

Corresponding author(s): Kenneth King-yip Cheng

Last updated by author(s): Oct 6, 2021

Reporting Summary

Nature Portfolio wishes to improve the reproducibility of the work that we publish. This form provides structure for consistency and transparency in reporting. For further information on Nature Portfolio policies, see our Editorial Policies and the Editorial Policy Checklist.

Statistics

For all statistical analyses, confirm that the following items are present in the figure legend, table legend, main text, or Methods section.

n/a	Confirmed	
☐	☒	The exact sample size (*n*) for each experimental group/condition, given as a discrete number and unit of measurement
☐	☒	A statement on whether measurements were taken from distinct samples or whether the same sample was measured repeatedly
☐	☒	The statistical test(s) used AND whether they are one- or two-sided<br>*Only common tests should be described solely by name; describe more complex techniques in the Methods section.*
☐	☒	A description of all covariates tested
☐	☒	A description of any assumptions or corrections, such as tests of normality and adjustment for multiple comparisons
☐	☒	A full description of the statistical parameters including central tendency (e.g. means) or other basic estimates (e.g. regression coefficient) AND variation (e.g. standard deviation) or associated estimates of uncertainty (e.g. confidence intervals)
☐	☒	For null hypothesis testing, the test statistic (e.g. *F*, *t*, *r*) with confidence intervals, effect sizes, degrees of freedom and *P* value noted<br>*Give P values as exact values whenever suitable.*
☒	☐	For Bayesian analysis, information on the choice of priors and Markov chain Monte Carlo settings
☒	☐	For hierarchical and complex designs, identification of the appropriate level for tests and full reporting of outcomes
☒	☐	Estimates of effect sizes (e.g. Cohen's *d*, Pearson's *r*), indicating how they were calculated

Our web collection on statistics for biologists contains articles on many of the points above.

Software and code

Policy information about availability of computer code

Data collection	Confocal images were obtained using Leica LAS X.
Data analysis	Data analysis was performed with ImageJ, FlowJo, GraphPad Prism 6.0 and IBM SPSS Statistics 26.0.

For manuscripts utilizing custom algorithms or software that are central to the research but not yet described in published literature, software must be made available to editors and reviewers. We strongly encourage code deposition in a community repository (e.g. GitHub). See the Nature Portfolio guidelines for submitting code & software for further information.

Data

Policy information about availability of data

All manuscripts must include a data availability statement. This statement should provide the following information, where applicable:

- Accession codes, unique identifiers, or web links for publicly available datasets
- A description of any restrictions on data availability
- For clinical datasets or third party data, please ensure that the statement adheres to our policy

The data generated in this study are provided in the Supplementary Information/Source Data file. Source data are provided with this paper.

Field-specific reporting

Please select the one below that is the best fit for your research. If you are not sure, read the appropriate sections before making your selection.

☒ Life sciences ☐ Behavioural & social sciences ☐ Ecological, evolutionary & environmental sciences

For a reference copy of the document with all sections, see nature.com/documents/nr-reporting-summary-flat.pdf

Life sciences study design

All studies must disclose on these points even when the disclosure is negative.

Sample size

Sample size for each experiment was based on literature documentation of similar well-characterized experiments and our previous publications. No statistical method was used to pre-determine sample size.
Selected references:
1. Cheng, K.K. et al. Adiponectin-induced endothelial nitric oxide synthase activation and nitric oxide production are mediated by APPL1 in endothelial cells. Diabetes 56, 1387-1394 (2007).
2. Cheng, K.K. et al. APPL1 potentiates insulin-mediated inhibition of hepatic glucose production and alleviates diabetes via Akt activation in mice. Cell Metab 9, 417-427 (2009).
3. Cheng, K.K. et al. APPL1 potentiates insulin secretion in pancreatic beta cells by enhancing protein kinase Akt-dependent expression of SNARE proteins in mice. Proc Natl Acad Sci U S A 109, 8919-8924 (2012).

Data exclusions

No inclusion or exclusion criteria were used.

Replication

For animal experiments, the experiment was performed once and each mouse was treated as a biological replicate. For other experiments, at least 3 technical replicates were included and each experiment was repeated 3 times to ensure reproducibility.

Randomization

For animal experiments, all animals were sex- and age-matched with the same genetic background and littermates were used. For bone marrow transplantation, C57BL/6N mice were randomly assigned to each group and transplanted with bone marrow from APPL1-KO mice and their WT littermates. Mice were grouped according to the genotype of the reconstituted bone marrow and no randomization was used. For in vitro experiments, no randomization was used and is grouped according to the genotype of the bone marrow-derived macrophages. Randomization is irrelevant as the experimental readout by equipment cannot be interfered.

Blinding

For immunohistological and histological analysis, the investigators were blinded to the sample identity. For other experiments, blinding is irrelevant as the experimental readout by the equipment cannot be interfered by the subjectivity of the researchers. For immunoblotting, quantification was performed with ImageJ for comparing protein expressions between samples to ensure objectivity.

Reporting for specific materials, systems and methods

We require information from authors about some types of materials, experimental systems and methods used in many studies. Here, indicate whether each material, system or method listed is relevant to your study. If you are not sure if a list item applies to your research, read the appropriate section before selecting a response.

Materials & experimental systems

n/a	Involved in the study
☐	☒ Antibodies
☐	☒ Eukaryotic cell lines
☒	☐ Palaeontology and archaeology
☐	☒ Animals and other organisms
☒	☐ Human research participants
☒	☐ Clinical data
☒	☐ Dual use research of concern

Methods

n/a	Involved in the study
☒	☐ ChIP-seq
☐	☒ Flow cytometry
☒	☐ MRI-based neuroimaging

Antibodies

Antibodies used

1. HSP90, #4874, Cell Signaling Technology
2. NLRP3, #15101, Cell Signaling Technology
3. NLRP3, #ab270449, Abcam
4. Caspase-1, #AG-20B-0042-C100, Adipogen
5. IL-1β, #12242, Cell Signaling Technology
6. Cleaved IL-1β, #52718, Cell Signaling Technology
7. Cleaved caspase-1, #67314, Cell Signaling Technology
8. Tom20, #42406, Cell Signaling Technology
9. Tom20, #sc-17764, Santa Cruz
10. β-actin, #sc-47778, Santa Cruz

11. LC3B, #12741, Cell Signaling Technology
12. Rab5, #46449, Cell Signaling Technology
13. APPL1, #3858, Cell Signaling Technology
14. APPL1, #sc-271909, Santa Cruz
15. F4/80, #MCA497RT, Bio-Rad
16. IκBα, #9242, Cell Signaling Technology
17. p-IκBα (ser32/36), #9241, Cell Signaling Technology
18. p65, #8242, Cell Signaling Technology
19. p-p65 (ser536), #3033, Cell Signaling Technology
20. LAMP1, #sc-20011, Santa Cruz
21. LAMP2A, #ab18528, Abcam
22. p62, #5114, Cell Signaling Technology
23. Cyt C, #4272, Cell Signaling Technology
24. AIM2, #sc-293174, Santa Cruz
25. Alexa Fluor® 647 Anti-TOMM20, #ab205487, Abcam
26. TGN38, #sc-166594, Santa Cruz
27. PE-F4/80, #123110, BioLegend
28. FITC-Cd11b, #101206, BioLegend
29. Goat anti-mouse IgG (H+L) Cross-adsorbed secondary antibody, Alexa Fluor 488, #A-11001, Invitrogen
30. Goat anti-rabbit IgG (H+L) Cross-adsorbed secondary antibody, Alexa Fluor 594, #A-11012, Invitrogen
31. Goat anti-rabbit IgG (H+L) Cross-adsorbed secondary antibody, Alexa Fluor 647, #A-21244, Invitrogen
32. Anti-rabbit IgG, HRP-linked antibody, #7074, Cell Signaling Technology
33. Anti-mouse IgG, HRP-linked antibody, #7076, Cell Signaling Technology

Validation

1. HSP90, #4874, Cell Signaling Technology. Cited by Lee P, Malik D, Perkons N, et al. Targeting glutamine metabolism slows soft tissue sarcoma growth. Nat Commun. 2020;11(1):498 and Rennhack JP, To B, Swiatnicki M, et al. Integrated analyses of murine breast cancer models reveal critical parallels with human disease. Nat Commun. 2019;10(1):3261.
2. NLRP3, #15101, Cell Signaling Technology. Cited by Kim SY, Jeong JM, Kim SJ, et al. Pro-inflammatory hepatic macrophages generate ROS through NADPH oxidase 2 via endocytosis of monomeric TLR4-MD2 complex. Nat Commun. 2017;8(1):2247 and Benmerzoug S, Rose S, Bounab B, et al. STING-dependent sensing of self-DNA drives silica-induced lung inflammation. Nat Commun. 2018;9(1):5226.
3. NLRP3, #ab270449, Abcam. Knockout validated by Abcam. Cited by Yue R, Zheng Z, Luo Y, et al. NLRP3-mediated pyroptosis aggravates pressure overload-induced cardiac hypertrophy, fibrosis, and dysfunction in mice: cardioprotective role of irisin. Cell Death Discov. 2021;7(1):50. and Dai W, Wang M, Wang P, et al. Lncrna neat1 ameliorates lps[illegible]induced inflammation in mg63 cells by activating autophagy and suppressing the nlrp3 inflammasome. Int J Mol Med. 2020;47(2):607-620.
4. Caspase-1, #AG-20B-0042-C100, Adipogen. Validated for western blotting with cell extract and sueprnatant from bone marrow-derived dendritic cells from wild-type, NLRP3-/- and caspase-1-/- mice activated or not by 5 µM Nigericin for 30 min. Proteins were separated by SDS-PAGE under reducing conditions, transferred to nitrocellulose and incubated with the antibody at 1µg/ml concentration. Proteins were visualized by a chemiluminescence detection system.
5. IL-1β, #12242, Cell Signaling Technology. Validated for western blotting using extracts from THP-1 cells with or without LPS (100 ng/ml) treatment for 3 hours. Cited by Zhang Y, Lv X, Hu Z, et al. Protection of Mcc950 against high-glucose-induced human retinal endothelial cell dysfunction. Cell Death Dis. 2017;8(7):e2941. and Márquez S, Fernández JJ, Terán-cabanillas E, et al. Endoplasmic Reticulum Stress Sensor IRE1α Enhances IL-23 Expression by Human Dendritic Cells. Front Immunol. 2017;8:639.
6. Cleaved IL-1β, #52718, Cell Signaling Technology. Validated for western blot analysis with recombinant mouse Interleukin-1β. Cited by Chu LH, Indramohan M, Ratsimandresy RA, et al. The oxidized phospholipid oxPAPC protects from septic shock by targeting the non-canonical inflammasome in macrophages. Nat Commun. 2018;9(1):996. and Wu C, Pan LL, Niu W, et al. Modulation of Gut Microbiota by Low Methoxyl Pectin Attenuates Type 1 Diabetes in Non-obese Diabetic Mice. Front Immunol. 2019;10:1733.
7. Cleaved caspase-1, #67314, Cell Signaling Technology. Cited by Kurundkar D, Kurundkar AR, Bone NB, et al. SIRT3 diminishes inflammation and mitigates endotoxin-induced acute lung injury. JCI Insight. 2019;4(1) and Zhao Y, Lu F, Ye J, et al. Myeloid-Derived Suppressor Cells and γδT17 Cells Contribute to the Development of Gastric MALT Lymphoma in -Infected Mice. Front Immunol. 2019;10:3104.
8. Tom20, #42406, Cell Signaling Technology. Cited by Laker RC, Drake JC, Wilson RJ, et al. Ampk phosphorylation of Ulk1 is required for targeting of mitochondria to lysosomes in exercise-induced mitophagy. Nat Commun. 2017;8(1):548 and Anderson GR, Wardell SE, Cakir M, et al. Dysregulation of mitochondrial dynamics proteins are a targetable feature of human tumors. Nat Commun. 2018;9(1):1677.
9. Tom20, #sc-17764, Santa Cruz. Cited by Tan K, Fujimoto M, Takii R, Takaki E, Hayashida N, Nakai A. Mitochondrial SSBP1 protects cells from proteotoxic stresses by potentiating stress-induced HSF1 transcriptional activity. Nat Commun. 2015;6:6580. and El-khattouti A, Selimovic D, Hannig M, et al. Imiquimod-induced apoptosis of melanoma cells is mediated by ER stress-dependent Noxa induction and enhanced by NF-κB inhibition. J Cell Mol Med. 2016;20(2):266-86.
10. β-actin, #sc-47778, Santa Cruz. Cited by Péladeau C, Adam N, Bronicki LM, et al. Identification of therapeutics that target eEF1A2 and upregulate utrophin A translation in dystrophic muscles. Nat Commun. 2020;11(1):1990. and Jones GG, Del río IB, Sari S, et al. SHOC2 phosphatase-dependent RAF dimerization mediates resistance to MEK inhibition in RAS-mutant cancers. Nat Commun. 2019;10(1):2532.
11. LC3B, #12741, Cell Signaling Technology. Cited by Péladeau C, Adam N, Bronicki LM, et al. Identification of therapeutics that target eEF1A2 and upregulate utrophin A translation in dystrophic muscles. Nat Commun. 2020;11(1):1990. and Vera-ramirez L, Vodnala SK, Nini R, Hunter KW, Green JE. Autophagy promotes the survival of dormant breast cancer cells and metastatic tumour recurrence. Nat Commun. 2018;9(1):1944.
12. Rab5, #46449, Cell Signaling Technology. Cited by Hu ZQ, Rao CL, Tang ML, et al. Rab32 GTPase, as a direct target of miR-30b/c, controls the intracellular survival of Burkholderia pseudomallei by regulating phagosome maturation. PLoS Pathog. 2019;15(6):e1007879. and Malik N, Nirujogi RS, Peltier J, et al. Phosphoproteomics reveals that the hVPS34 regulated SGK3 kinase specifically phosphorylates endosomal proteins including Syntaxin-7, Syntaxin-12, RFIP4 and WDR44. Biochem J. 2019;476(20):3081-3107.
13. APPL1, #3858, Cell Signaling Technology. Cited by Sneeggen M, Pedersen NM, Campsteijn C, Haugsten EM, Stenmark H, Schink KO. WDFY2 restrains matrix metalloproteinase secretion and cell invasion by controlling VAMP3-dependent recycling. Nat Commun. 2019;10(1):2850. and Koike S, Jahn R. SNAREs define targeting specificity of trafficking vesicles by combinatorial interaction with

tethering factors. Nat Commun. 2019;10(1):1608.
14. APPL1, #sc-271909, Santa Cruz. KO validated in our lab using APPL1 knockout bone marrow-derived macrophages. Immunoblotting was performed using this primary antibody for detection of APPL1 in bone marrow-derived macrophages from APPL1 knockout and wild-type control mice. Samples from knockout mice did not show any band when samples from wild-type mice displayed protein band at the predicted molecular weight.
15. F4/80, #MCA497RT, Bio-Rad. Cited by Schulz C, Gomez perdiguero E, Chorro L, et al. A lineage of myeloid cells independent of Myb and hematopoietic stem cells. Science. 2012;336(6077):86-90. and Kierdorf K, Erny D, Goldmann T, et al. Microglia emerge from erythromyeloid precursors via Pu.1- and Irf8-dependent pathways. Nat Neurosci. 2013;16(3):273-80.
16. IκBα, #9242, Cell Signaling Technology. Cited by Li F, Li Y, Liang H, et al. HECTD3 mediates TRAF3 polyubiquitination and type I interferon induction during bacterial infection. J Clin Invest. 2018;128(9):4148-4162. and Wu Y, Du S, Johnson JL, et al. Microglia and amyloid precursor protein coordinate control of transient Candida cerebritis with memory deficits. Nat Commun. 2019;10(1):58.
17. p-IκBα (ser32/36), #9241, Cell Signaling Technology. Cited by Lee SW, Park Y, So T, et al. Identification of regulatory functions for 4-1BB and 4-1BBL in myelopoiesis and the development of dendritic cells. Nat Immunol. 2008;9(8):917-26. and Karki R, Man SM, Malireddi RKS, et al. NLRC3 is an inhibitory sensor of PI3K-mTOR pathways in cancer. Nature. 2016;540(7634):583-587.
18. p65, #8242, Cell Signaling Technology. Cited by Kuwahara M, Suzuki J, Tofukuji S, et al. The Menin-Bach2 axis is critical for regulating CD4 T-cell senescence and cytokine homeostasis. Nat Commun. 2014;5:3555. and Grabner B, Schramek D, Mueller KM, et al. Disruption of STAT3 signalling promotes KRAS-induced lung tumorigenesis. Nat Commun. 2015;6:6285.
19. p-p65 (ser536), #3033, Cell Signaling Technology. Cited by Sindi HA, Russomanno G, Satta S, et al. Therapeutic potential of KLF2-induced exosomal microRNAs in pulmonary hypertension. Nat Commun. 2020;11(1):1185. and Choi I, Zhang Y, Seegobin SP, et al. Microglia clear neuron-released α-synuclein via selective autophagy and prevent neurodegeneration. Nat Commun. 2020;11(1):1386.
20. LAMP1, #sc-20011, Santa Cruz. Cited by Su X, Yu Y, Zhong Y, et al. Interferon-γ regulates cellular metabolism and mRNA translation to potentiate macrophage activation. Nat Immunol. 2015;16(8):838-849. and Kasper L, König A, Koenig PA, et al. The fungal peptide toxin Candidalysin activates the NLRP3 inflammasome and causes cytolysis in mononuclear phagocytes. Nat Commun. 2018;9(1):4260.
21. LAMP2A, #ab18528, Abcam. Cited by Takeda S, Wegmann S, Cho H, et al. Neuronal uptake and propagation of a rare phosphorylated high-molecular-weight tau derived from Alzheimer's disease brain. Nat Commun. 2015;6(1):8490. and Magalhães-Novais S, Bermejo-Millo JC, Loureiro R, et al. Cell quality control mechanisms maintain stemness and differentiation potential of P19 embryonic carcinoma cells. Autophagy. 2020;16(2):313-333.
22. p62, #5114, Cell Signaling Technology. Cited by Harhouri K, Navarro C, Depetris D, et al. MG132-induced progerin clearance is mediated by autophagy activation and splicing regulation. EMBO Mol Med. 2017;9(9):1294-1313. and Jena KK, Kolapalli SP, Mehto S, et al. TRIM16 controls assembly and degradation of protein aggregates by modulating the p62-NRF2 axis and autophagy. EMBO J. 2018;37(18).
23. Cyt C, #4272, Cell Signaling Technology. Cited by Zhang Y, Kim MS, Jia B, et al. Hypothalamic stem cells control ageing speed partly through exosomal miRNAs. Nature. 2017;548(7665):52-57. and Pan J-X, Tang F, Xiong F, et al. APP promotes osteoblast survival and bone formation by regulating mitochondrial function and preventing oxidative stress. Cell Death Dis. 2018;9(11):1077.
24. AIM2, #sc-293174, Santa Cruz. Cited by Gao J, Peng S, Shan X, et al. Inhibition of AIM2 inflammasome-mediated pyroptosis by Andrographolide contributes to amelioration of radiation-induced lung inflammation and fibrosis. Cell Death Dis. 2019;10(12):957.
25. Alexa Fluor® 647 Anti-TOMM20, #ab205487, Abcam. Cited by Park H, Choi D, Park JS, et al. Scalable and isotropic expansion of tissues with simply tunable expansion ratio. Adv Sci. 2019;6(22):1901673.
26. TGN38, #sc-166594, Santa Cruz. Cited by Knupp A, Mishra S, Martinez R, et al. Depletion of the ad risk gene sorl1 selectively impairs neuronal endosomal traffic independent of amyloidogenic app processing. Cell Reports. 2020;31(9):107719. and Vogelgesang S, Niebert S, Renner U, et al. Analysis of the serotonergic system in a mouse model of rett syndrome reveals unusual upregulation of serotonin receptor 5b. Front Mol Neurosci. 2017;10.
27. PE-F4/80, #123110, BioLegend. Cited by Doni A, Parente R, Laface I, et al. Serum amyloid P component is an essential element of resistance against Aspergillus fumigatus. Nat Commun. 2021;12(1):3739 and Wei Z, Zhang X, Yong T, et al. Boosting anti-PD-1 therapy with metformin-loaded macrophage-derived microparticles. Nat Commun. 2021;12(1):440.
28. FITC-Cd11b, #101206, BioLegend. Cited by Wei Z, Zhang X, Yong T, et al. Boosting anti-PD-1 therapy with metformin-loaded macrophage-derived microparticles. Nat Commun. 2021;12(1):440. and Yang W, Yu T, Huang X, et al. Intestinal microbiota-derived short-chain fatty acids regulation of immune cell IL-22 production and gut immunity. Nat Commun. 2020;11(1):4457.

Eukaryotic cell lines

Policy information about cell lines

Cell line source(s)	NCTC clone 929 [L cell, L-929, derivative of Strain L] (ATCC® CCL-1™) from ATCC.
Authentication	Cell line used in this study was not authenticated.
Mycoplasma contamination	The cell line was not tested for mycoplasma contamination.
Commonly misidentified lines (See ICLAC register)	No commonly misidentified line was used in this study.

Animals and other organisms

Policy information about studies involving animals; ARRIVE guidelines recommended for reporting animal research

Laboratory animals	Male APPL1 knock out mice and their wild-type controls with C57BL/6N genetic background were used in this study at 10 weeks of age. The animals used in this study were sex- and age-matched.
Wild animals	No wild animal was used.
Field-collected samples	No field-collected sample was included in this study.

Ethics oversight	All animal experimental protocols were approved by Animal Subjects Ethics Sub-Committee at The Hong Kong Polytechnic University and the Committee on the Use of Live Animals in Teaching and Research at The University of Hong Kong.

Note that full information on the approval of the study protocol must also be provided in the manuscript.

Flow Cytometry

Plots

Confirm that:

☒ The axis labels state the marker and fluorochrome used (e.g. CD4-FITC).

☒ The axis scales are clearly visible. Include numbers along axes only for bottom left plot of group (a 'group' is an analysis of identical markers).

☒ All plots are contour plots with outliers or pseudocolor plots.

☒ A numerical value for number of cells or percentage (with statistics) is provided.

Methodology

Sample preparation	After the treatment indicated in the figure legends, cell culture supernatant was removed and bone marrow-derived macrophages were washed with PBS twice. Cells were then detached from non-tissue culture dishes using cell dissociation buffer (#13151014, Thermofisher) and centrifuged at 500 g at 4 degree for 5 minutes. The cell pellet was resuspended in PBS with 1 mM EDTA, 25 mM HEPES, 1% FBS and kept on ice in dark until analysis.
Instrument	BD FACSAria™ III with blue (488 nm), red (633 nm), yellow green (561 nm), violet (405 nm) and near-UV (375 nm) lasers.
Software	FlowJo.
Cell population abundance	No cell sorting was used in this study.
Gating strategy	To differentiate between positive and negative population, isotype controls were used to stain the cells and define the gate for positive population. For mt-Keima experiment, cells infected with the control adenovirus were used as negative control to define the gate for mt-Keima expressing cells.

☒ Tick this box to confirm that a figure exemplifying the gating strategy is provided in the Supplementary Information.
